# Detection of unwarranted CT radiation exposure from patient and imaging protocol meta-data using regularized regression

**DOI:** 10.1016/j.ejro.2019.04.007

**Published:** 2019-06-05

**Authors:** Ruidi Chen, Ioannis Ch. Paschalidis, Hiroto Hatabu, Vladimir I. Valtchinov, Jenifer Siegelman

**Affiliations:** a Center for Evidence-Based Imaging (CEBI), Brigham and Women’s Hospital, United States; b Department of Radiology, Brigham and Women’s Hospital, Harvard Medical School, United States; c Department of Biomedical Informatics, Harvard Medical School, United States; d Department of Biomedical Engineering, Boston University, United States; e Department of Electrical and Computer Engineering, Boston University, 8 St. Mary’s Street, Boston, MA 02215, USA; f Takeda Pharmaceuticals, United States

**Keywords:** Outlier detection, CT radiation dose safety, Regression model

## Abstract

**Background:**

Variability in radiation exposure from CT scans can be appropriate and driven by patient features such as body habitus. Quantitative analysis may be performed to discover instances of unwarranted radiation exposure and to reduce the probability of such occurrences in future patient visits. No universal process to perform identification of outliers is widely available, and access to expertise and resources is variable.

**Objective:**

The goal of this study is to develop an automated outlier detection procedure to identify all scans with an unanticipated high radiation exposure, given the characteristics of the patient and the type of the exam.

**Materials and methods:**

This Institutional Review Board-approved retrospective cohort study was conducted from June 30, 2012 - December 31, 2013 in a quaternary academic medical center. The de-identified dataset contained 28 fields for 189,959 CT exams. We applied the variable selection method Least Absolute Shrinkage and Selection Operator (LASSO) to select important variables for predicting CT radiation dose. We then employed a regression approach that is robust to outliers, to learn from data a predictive model of CT radiation doses given important variables identified by LASSO. Patient visits whose predicted radiation dose was statistically different from the radiation dose actually received were identified as outliers.

**Results:**

Our methodology identified 1% of CT exams as outliers. The top-5 predictors discovered by LASSO and strongly correlated with radiation dose were Tube Current, kVp, Weight, Width of collimator, and Reference milliampere-seconds. A human expert validation of the outlier detection algorithm has yielded specificity of 0.85 [95% CI 0.78-0.92] and sensitivity of 0.91 [95% CI 0.85-0.97] (PPV = 0.84, NPV = 0.92). These values substantially outperform alternative methods we tested (F1 score 0.88 for our method against 0.51 for the alternatives).

**Conclusion:**

The study developed and tested a novel, automated method for processing CT scanner meta-data to identify CT exams where patients received an unwarranted amount of radiation. Radiation safety and protocol review committees may use this technique to uncover systemic issues and reduce future incidents.

## 1 Introduction

Good clinical practice and regulatory compliance include routine performance of CT protocol review by a multidisciplinary team of experts. Review and quantitative analysis of retrospective patient imaging exams and protocol meta-data is typically performed to discover instances of unwarranted radiation exposure. Further analysis of these instances can help identify systemic problems and reduce the probability of such occurrences in future patient visits. Multiple authors have reported on methods to review and manage protocols [[1], [2], [3]]. Other studies have described approaches using non-adherence protocol review [4], manual root cause analysis [5], process control methods [6,7] and in children, a size-based quality informed framework [8]. Related work studied the effects of transitioning to digital image acquisition [9] and estimated collective radiation dose due to medical exams in an entire population [10].

This work is motivated by a desire to identify exposure events which may be missed by traditional outlier detection methods. Such methods “flag” cases where the radiation dose exceeded an average value by several multiples of the standard deviation. In the absence of any knowledge on which cases may be outliers, computing an average from past data that do contain some outliers has the effect of skewing the average; and thus, masking future outliers. We report on the development and validation of an automated method for detecting radiation exposure outliers, using only two data types: patient demographics and protocol meta-data. The key innovation is the development of a predictive model of appropriate radiation exposure; outliers are then identified by statistically large deviations of the actual radiation dose from the predicted value.

The developed method is based on a Regularized Regression (RR) [11] predictive model. By design, the model is robust to being exposed to outliers during training, which is not the case with more standard regression approaches. The most relevant predictors are extracted using Least Absolute Shrinkage and Selection Operator (LASSO) [12], a penalized regression model that induces sparsity on the predictor level and applies well to cases where the number of predictors far exceeds the number of patients. This type of sparse regression model can isolate relatively few predictive factors and, thus, enable interpretation of the predictions.

## 2 Materials and methods

### 2.1 Study design and setting

This HIPAA-compliant, Institutional Review Board (IRB)-approved retrospective cohort study was conducted at an academic medical system including a 793-bed quaternary care hospital, and two outpatient imaging facilities. The IRB did not require informed consent. All consecutive CT examinations performed on the 11 operational CT scanners (GE, Philips, and Siemens) were eligible for study inclusion between June 30, 2012 and December 31, 2013, a time-period during which there were no scanner equipment or software changes.

### 2.2 Data description and pre-processing

We followed the steps below to pre-process the data.

• Examination metadata were de-identified at the site.
• Patient visits with more than half of the corresponding variables missing, or a missing value for CT Dose Index (CTDI), or a missing value for patient weight, are discarded.
• CT examinations of the body were retained. Head CT examinations were not included.
• Binary encoding: categorical variables (such as scanner type, protocol type, patient gender, X-ray modulation type) are encoded using binary (indicator) variables for each category. Furthermore, categories present only in a small number of exams are deleted.
• Variables that have low correlation with CTDI (absolute value below 0.06) are removed from further consideration, e.g., scan length, the number of scans, age, pitch factor, and the number of X-ray sources, as the unit of analysis for this method is the single acquisition event rather than the total patient encounter. The specific threshold 0.06 is selected because it separates the variables with relatively high correlation with CTDI (0.30 on average) from the ones with low correlation (0.01 on average).
• We impute the missing values by the mean (for numerical predictors) or mode (for categorical predictors).
• Normalization of the predictors: all predictors are standardized by subtracting the mean and dividing by the standard deviation.
• Splitting the data into a training set and a test set randomly: as is common in machine learning, the dataset of CT exams is randomly split into a training and a test set. The training set is used to train the model and the test set to evaluate its performance. Since from a statistical point of view, all the data points (patients’ features) are drawn from the same distribution, we do not differentiate between patients whose records appear earlier in time than others with later time stamps.

### 2.3 Outcome measures

CT Dose Index (CTDI_vol_), which measures the amount of exposure to CT radiation for each dose event, was chosen as the primary outcome measure (dependent variable). Note that this is a metric of the conformance of the protocol and the behavior of the automatic exposure control on a per acquisition level. Multi-phase evaluations are included, but the number of phases and/or the repetition of acquisition events are beyond the scope of this method of analysis. In addition, we use the coefficient of determination, R [2], to measure the goodness of fit for the regression procedure, which assesses its ability to accurately predict CTDI_vol._ Finally, we use the accuracy of the outlier detection procedure, expressed in terms of sensitivity and specificity, by employing a human expert to assess whether outliers identified by the algorithms are indeed outliers or not.

### 2.4 Statistical analysis

#### 2.4.1 Statistical objective and background

**Fig. 1:**
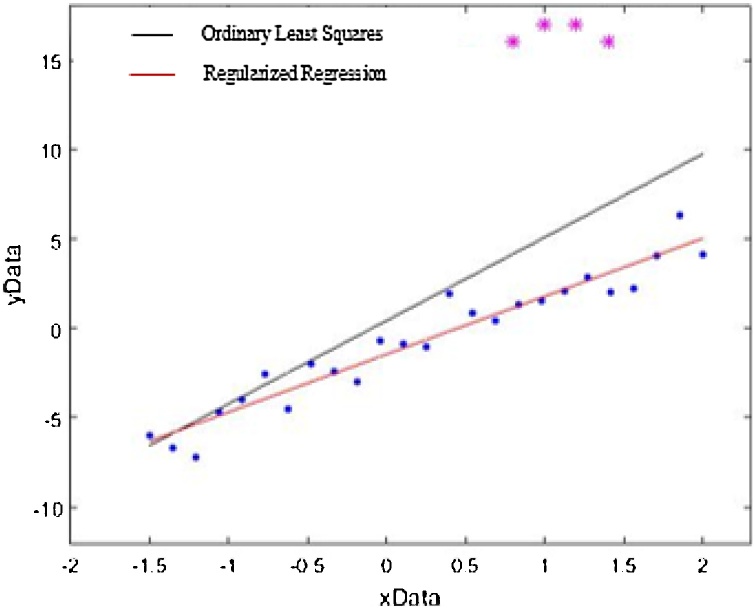
Comparison between OLS and Regularized Regression.

The variables containing patient characteristics such as age, gender and weight, and CT exam-related variables such as scanner type, X-ray modulation type, exposure time, the number of X-ray sources, and tube current, etc., are captured by a predictor vector $x\in R^{d}$, for each exam i=1,…,N. We are interested in predicting the normal amount of exposure to CT radiation (CTDI) given the predictors, and “screening out” the outliers, which are defined to be the exams where the patient received an abnormal radiation dose. We denote the CTDI by a continuous variable y∈R. Using statistical terminology, the problem can be cast as a regression problem. We seek to find a regression plane that accurately describes the relationship between the CTDI y and the predictors x for non-outlying CT exams. Ordinary Least Squares (OLS) regression cannot accomplish this goal due to the presence of outliers. For example, in Fig. 1, we plot (blue) sample points (x, y) according to some probability distribution that satisfies a roughly linear relationship. Suppose now our samples include few outliers (magenta) that are unknown to us. OLS produces the black line; shifting the “true” line to accommodate the outliers. When we use regression residuals to detect outliers, it can be seen that moderate outliers may not be identified as they would not be too far from the black line. What is needed is the line that is robust to outliers, as indicated by the red line in Fig. 1 and such a line can be computed using a robust, Regularized Regression (RR) technique [11].

#### 2.4.2 The LASSO + RR framework

A set of predictors is needed to perform the regression analysis. Usually in medical datasets, the number of predictors far exceeds the number of patients, which will cause a rank deficiency issue commonly seen in OLS analysis. To solve this problem, an effective variable selection method called Least Absolute Shrinkage and Selection Operator (LASSO) [12] is applied to extract a subset of effective predictors. Earlier work has established that a sparse regression model enhances the predictive power of the regression model and produces more interpretable results [[13], [14], [15]].

With the effective predictors x obtained from LASSO, we use a Regularized Regression (RR) model to learn a robustified relationship between the CTDI y and. The mechanism is as follows: we minimize the deviation of y from the prediction produced by the model, plus a term that penalizes the complexity of the model. It has been established [11] that this penalty term provides protection against outliers, essentially producing a regression model that fits the non-outlying training samples.

Given such a robust regression model, outliers are identified when the absolute residuals from RR, i.e., the absolute value of the difference between y and the prediction, exceeds 3 times the standard deviation of the residual.

We use the metric $R^{2}$, the coefficient of determination in statistics, to evaluate how well the regression model predicts CTDI using the selected predictors.

$$R^{2}≜1-\frac{the\;sum\;of\;squared\;residuals\;}{N\text{*}the\;variance\;of\;CTDI},$$

where N is the total number of exams considered, and the residual for each exam is the difference between the CTDI and the prediction. $R^{2}$ assesses the percentage of variation in CTDI that is explained by the model.

Analyses and visualizations were performed using the R statistical programming environment (version 3.2.2) using the glmnet R package for LASSO modelling [16].

#### 2.4.3 Outlier algorithm validation process

To assess the accuracy of the outlier cohort discovery process, we conducted a manual validation in which the results of a human-expert classification were compared to those extracted by the algorithm. One radiologist (a body imaging radiologist with 14 years of experience) has performed a manual chart and image review of each of the outliers identified and cases which were classified as appropriate, blinded to the results of the algorithm. A validation sample size of a random 200 sample cases were reviewed, in accordance with a power calculation of a standard significance level (alpha) of 5% and power (1-beta) of 80% [17]. Specificity, sensitivity and 95% Confidence Intervals (95% CIs) of the algorithmic outlier detection method were computed.

#### 2.4.4 Alternative methods

We compared our outlier detection method against two alternatives on the same validation set of 200 samples that were reviewed by the human expert. The first alternative method is what we call a “cutoff” method. We compute the average and standard deviation of CTDI over a training set and identify as outliers exams where the CTDI was larger than the average plus 3 times the standard deviation.

The second alternative method was identical with our approach, except that Ordinary Least Squares (OLS) was used in lieu of the RR method. As with our method, the regression residuals (this time from OLS) were used to detect outliers.

## 3 Results

### 3.1 Study cohort

The original de-identified dataset contained 28 fields for 189,959 CT exams, and the per acquisition CT Dose Index (CTDI), which measures the amount of exposure to CT radiation. Mean patient age was 60.6 ± 17.1 years; 54.7% were females.

### 3.2 Pre-processing and variable selection

After pre-processing, we are left with 606 numerically encoded predictors for 88,566 CT exams. Patient characteristics for these exams are summarized in Table 1.

**Table 1:** Patient characteristics for exams after pre-processing.

Characteristic	Group	Percentage, % (N = 88,566)	Mean	Standard Deviation
**Gender**	Female	54.10	NA	
	Male	45.90		
**Weight (kg)**	S (<56.5)	10.65	50.52	4.90
	M (≥56.5 and <79.5)	45.73	68.72	6.55
	L (≥79.5 and <102.5)	33.38	88.98	6.49
	XL (≥102.5 and <136.5)	8.98	113.69	8.95
	XXL (≥136.5)	1.26	156.19	26.81
**Age**	≤18	0.03	17	1.09
	>18 and ≤34	6.09	29	3.40
	>34 and ≤49	11.95	43	4.31
	>49 and ≤59	18.51	55	2.81
	>59 and ≤69	24.70	65	2.88
	>69 and ≤79	23.07	74	2.82
	>79 and ≤89	11.55	84	2.81
	>89	4.10	98	11.61

**Fig. 2:**
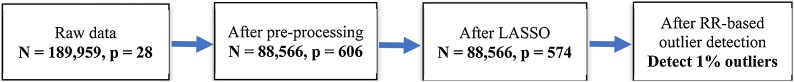
Schematic representation of the data pre-processing and analysis steps in the proposed LASSO + RR-based pipeline. N denotes the number of CT exams used and p the number of features (variables) retained for each exam.

The implementation of LASSO + RR is described in Fig. 2, where N is the number of patients, and p is the number of predictors. The figure illustrates the various steps in the implemented pipeline for outlier detection.

We found that 574 out of the 606 predictors (95%) were selected by the process described above. In Table 2 we summarize the most important predictors identified by the LASSO algorithm. Specifically, we run LASSO five times, each with a different training dataset, average the coefficients corresponding to the various predictors over the five runs, and report the predictors with the largest average coefficients. Note that all predictors are standardized, and thus it is reasonable to identify important predictors based on the magnitudes of the average coefficients. We do not list the categorical predictors in Table 2 since one single categorical variable may correspond to hundreds of numerically encoded variables (“dummy” variables).

**Table 2:** Important features identified by LASSO.

Important Predictors for CTDI_vol_ (ranked by the average LASSO coefficients)	Average coefficient
Tube Current	3.49
kVp	1.73
Weight	1.15
Width of collimator	0.66
Reference mAs	0.37

**Fig. 3:**
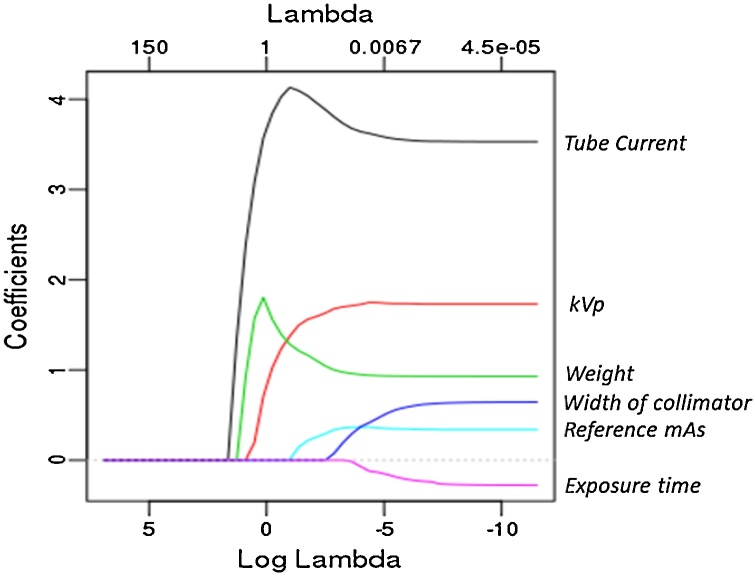
The coefficients’ paths for numerical predictors in LASSO. Lambda refers to the coefficient of the sparsity-inducing penalty in LASSO. A collimator is a metallic barrier with an aperture of variable width used to control the diameter of the X-ray beam.

**Fig. 4:**
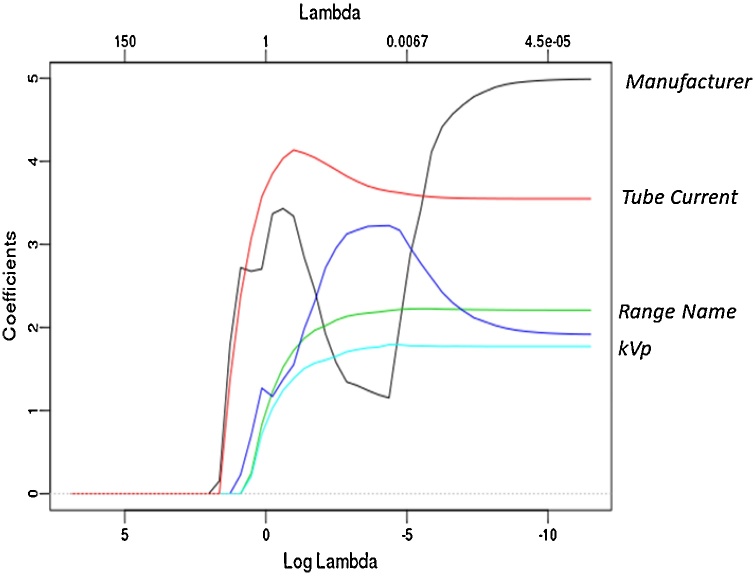
The coefficients’ paths for the top 5 predictors in LASSO. Range Name is a parameter specified by the protocol as to which region is being scanned in a multiple body part evaluation.

We also plot the coefficients for numerical predictors vs. the LASSO penalty parameter (Lambda) in Fig. 3. We see that the relative importance of the predictors implied by Fig. 3 coincides with Table 2. It is of interest to see which dummy variables are selected by LASSO to have a rough sense of the relative importance of the categorical predictors. In Fig. 4 we plot the coefficients’ paths for the top 5 predictors, 2 of which are the aforementioned numerical variables, and the rest represent the categorical variables such as manufacturer and range name. (The green and blue lines represent two different range names.)

### 3.3 Regularized Regression-based outlier detection and human expert validation

Given the effective predictors obtained by LASSO, we are able to fit the RR model, whose residuals are used to identify outliers. Our methodology identifies 1% patient visits as outliers. In addition, the percentage of the variance explained by the model is $R^{2}=76\text{\%}.$

Table 3 lists the results of the human expert validation of the outlier detection algorithm. The first row of the table lists how manual review classified the outliers identified by the algorithm, whereas the second row lists the results of manual review for the CT exams the algorithm classified as non-outliers. The specificity was at 0.85 [95% CI 0.78-0.92] and sensitivity was at 0.91 [95% CI 0.85-0.97] (Positive Predictive Value PPV = 0.84, Negative Predictive Value NPV = 0.92).

**Table 3:** Validation results of our RR-based outlier detection algorithm against manual review for 200 randomly selected entries.

		Manual Review		
		**True Outlier**	**False Outlier**	
**Algorithm**	**True Outlier**	84	16	100
	**False Outlier**	8	92	100
		92	108	

### 3.4 Comparison with alternative methods

We compared our outlier detection method against the two alternatives described earlier (cutoff method and OLS) on the validation set of 200 samples that were reviewed by the human expert.

Table 4, Table 5 present results similar to Table 3 but for the cutoff and the OLS method, respectively. Table 6 summarizes the accuracy of the three methods on the 200 human-reviewed validation dataset. For each method, we report sensitivity, specificity, PPV, NPV, and the F1 score, which is the harmonic mean of PPV and sensitivity.

**Table 4:** Validation results of the cutoff method against the 200 manually reviewed entries.

		Manual Review		
		**True Outlier**	**False Outlier**	
**Algorithm**	**True Outlier**	34	7	41
	**False Outlier**	58	101	159
		92	108

**Table 5:** Validation results of the OLS method against the 200 manually reviewed entries.

		Manual Review		
		**True Outlier**	**False Outlier**	
**Algorithm**	**True Outlier**	33	5	38
	**False Outlier**	59	103	162
		92	108

**Table 6:** Summary comparison of our proposed RR-based method against OLS and the cutoff method.

	Sensitivity	Specificity	PPV	NPV	F_1_ score
**RR**	0.91	0.85	0.84	0.92	0.88
**OLS**	0.36	0.95	0.87	0.64	0.51
**Cutoff**	0.37	0.94	0.83	0.64	0.51

For an additional point of comparison between our RR-based method and OLS, we considered the top-40 outliers identified by each method. Among these outliers, 7 of the top-40 OLS outliers (17.5%) were considered to be “false positives;” while all the top-40 outliers detected by our method were real outliers.

**Fig. 5:**
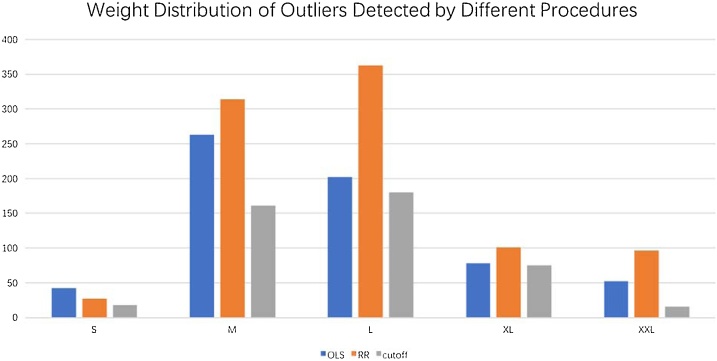
Outliers identified in the entire dataset (after pre-processing, **N = 88,566**) by the three methods, Cutoff, OLS, and our proposed RR-based method.

Finally, Fig. 5 plots the number of outliers detected by the three methods among the 88,566 CT exams maintained after the pre-processing steps.

## 4 Discussion

This work proposes a completely automated outlier detection method to identify CT scans during which the patient received an unusually high radiation dose. The developed method was able to identify outlying CT exams that were largely in agreement with assessment by a human expert, achieving specificity of 85% and sensitivity of 91%. After pre-processing of the original dataset, we evaluated 88,566 CT exams and identified 1% as outliers.

A regression analysis was used to train a predictive model of CT radiation dose given patient/exam characteristics. The gap between the actual radiation dose (as recorded in the dataset) and the value predicted by the regression model was used to detect outlying CT exams.

We compared our outlier detection method against two alternatives, a basic cutoff method and a method based on OLS regression in lieu of our Regularized Regression (RR)-based method. Comparing Table 3, Table 4, Table 5, it is evident that our method is in much better agreement with the human expert. Table 6 summarizes the key difference: our method has a much higher sensitivity with a corresponding high specificity. OLS and the cutoff methods detect much fewer exams as outliers (lower sensitivity). They have a higher specificity, but that is a natural consequence of very low sensitivity (i.e., the fewer outliers one detects, the fewer will be the false outliers). Figure 6 depicts the number of outliers detected by each method across the patient weight range (from Small to XXL, cf. weight ranges from Table 1). This figure confirms that we detect more outliers than the alternatives, especially for M and L patients who make up the majority of the patients (79.11%).

These results suggest that OLS regression fails to capture an accurate relationship between variables when the data are contaminated by outliers. The same is true for the simpler cutoff method. In contrast, our Regularized Regression (RR) approach demonstrates robustness to the presence of outliers in the training set. The effective predictors provided as input to RR were extracted using the Least Absolute Shrinkage and Selection Operator (LASSO) [12], a penalized regression model that induces sparsity of the predictors. This type of sparse regression model can reveal relatively few predictive factors and, thus, enable interpretation of the predictions.

Table 2 and Fig. 3, Fig. 4 reveal the most important predictors selected by LASSO, which include Tube Current, kVp, Weight, Width of collimator, and Reference mAs. The tube current is mathematically related to the calculation (estimate) of CTDIvol [18], and is thus expected to be one of the most effective predictors. The peak kilovoltage (kVp) has a positive non-linear relationship with CTDIvol [19].

Scan protocols are set in advance of any patient encounter. A reference set of values are entered into the scanner for a "standard size subject,” expressed as a reference milliampere seconds (Reference mAs). The exposure given to any given patient is the result of a response by the Automatic Exposure Control (AEC, a system setting which is modifiable). In certain protocols, reference mAs is purposely set to a high level by the physician, technologist and the physicist who designs the protocol, because they desire a higher low contrast detectability or thin slices viewing is needed to make the diagnosis. Aberrant patient positioning [20], or shields [21] or external hardware in the field of view when the scan is planned can alter the imaging systems AEC response to this selected parameter unexpectedly.

Low kVp studies are deliberately used to optimize the visualization of iodine, and typically used in vascular imaging. The trend identified vascular examinations to have low kVP. To improve penetration of the x-rays through larger size patients and produce an image, higher kVP may be required due to physical limitations of x-ray tubes. The automatic selection of a higher kVp may occur when the technical capacity of the tube to generate adequate milliamperes for the necessary scan time is not adequate. For vascular exams, selecting a higher than average kVp is problematic due to the reduction of contrast to noise ratio that is intrinsic in higher kVp imaging. For osseous imaging a higher kVp is often selected. These protocols are infrequent though and as such may not have undergone as careful systematic scrutiny by protocol review committees.

Some of the results reported in our variable selection and RR building steps have been previously independently identified by other studies. For example, we found that patient age is removed in the initial pre-processing step (see Materials and Methods) since it displays a low (below the cutoff threshold chosen in this study) correlation with the CTDI_vol_ predictor variable. Similar behavior has been reported in two recent studies [22,23]. Sodickson et al. have investigated the effects of patient size on radiation dose reduction and image quality in low-kVp CT pulmonary angiography, and have found patient age to be of no statistical significance as a factor correlated with radiation dose accumulation in CT pulmonary angiography [22]. More recently, Valtchinov et al. [23] have investigated the probability of a CT Dose-Check alarm to be generated when static CTDIvol-valued threshold was used to define what event constitutes an outlier. They have utilized a logistic regression formalism and found patient age to not be statistically significantly associated with predicting the probability of an exam being an outlier. Interestingly however, they have included an interaction term in their regression model of the form ***age*** *x* ***weight***, which did present a statistical significance in predicting an alarm even though the magnitude of the interaction term was quite small.

These two works have also confirmed that the patient weight is a statistically significant predictor variable for various metrics associated with radiation dose accumulation [22,23], which is consistent with our results.

The methodology we developed can readily leverage additional variables for each CT exam. In principle, any information available in the Radiation Dose Structured Report (RDSR) using the DICOM standard and available in most modern CT scanners could be used. Useful additional variables may include the use of a dual energy protocol and the size of the focal spot. For specific types of CT exams, such as cardiac CT, additional information such as the choice of gating techniques (prospective or retrospective) could also be incorporated. The use of additional variables has the potential to further improve the accuracy of the CTDI predictive model and, as a result, the performance of the outlier detection method.

Our study has limitations. One limitation of our data set was that scanners were limited to hospital CT units and software installed before 2012. Since then, devices and associated software have evolved. Taken together with the fact that the data set we used for building and testing our outlier prediction model has come from a quaternary care academic medical center, this limits the immediate claims of generalizability of our findings to other healthcare settings, scanner vendors and software versions. Therefore, it is highly desirable to independently test our algorithms on data from different institutions that might have a different mix of scanned vendors, imaging protocols and settings.

## 5 Conclusion

LASSO-based variable selection followed by a Regularized Regression using the selected set of variables is capable of accurately pinpointing the set of relevant dose outliers in an unsupervised-learning fashion. Because the method detects outliers when a predicted radiation dose is statistically different from the actual dose, it is capable of identifying outliers across the patient weight and size spectrum, outperforming more standard approaches that become “skewed” by the presence of outliers in the training data. We demonstrated that the proposed method is in excellent agreement with a human expert (85% specificity and 91% sensitivity).

The proposed method is a good candidate for being implementing in the workflow of medical imaging centers. Since it “flags” a small percentage of CT exams as outliers (1% in our study), it facilitates the work of radiation safety committees which will have a small percentage of cases to manually review. Review of these cases may help to identify systemic issues to address, leading to reduced future overexposure incidents.

The automated outlier detection procedure may also be implemented in a Radiology Information Systems (RIS) which is often used in conjunction with a Picture Archiving and Communication System (PACS). Such platforms have rudimentary CT dose monitoring modules that allow for computation of simple averages (e.g., of DLP) [24]. One could envision incorporating into these modules our outlier detection algorithm.

## Conflict of interest statement

The authors declare no conflict of interest.
